# Impact of Within-Tree Organ Distances on Floral Induction and Fruit Growth in Apple Tree: Implication of Carbohydrate and Gibberellin Organ Contents

**DOI:** 10.3389/fpls.2019.01233

**Published:** 2019-10-21

**Authors:** Fares Belhassine, Sébastien Martinez, Sylvie Bluy, Damien Fumey, Jean-Jacques Kelner, Evelyne Costes, Benoît Pallas

**Affiliations:** ^1^UMR AGAP, Univ. Montpellier, INRA, CIRAD, Montpellier SupAgro, Montpellier, France; ^2^ITK, Montpellier, France

**Keywords:** leaf/fruit removal, floral induction, fruit weight, non-structural carbohydrates, source-sink relationships, shoot/branch autonomy, Malus × domestica Borkh

## Abstract

In plants, organs are inter-dependent for growth and development. Here, we aimed to investigate the distance at which interaction between organs operates and the relative contribution of within-tree variation in carbohydrate and hormonal contents on floral induction and fruit growth, in a fruit tree case study. Manipulations of leaf and fruit numbers were performed in two years on “Golden delicious” apple trees, at the shoot or branch scale or one side of Y-shape trees. For each treatment, floral induction proportion and mean fruit weight were recorded. Gibberellins content in shoot apical meristems, photosynthesis, and non-structural carbohydrate concentrations in organs were measured. Floral induction was promoted by leaf presence and fruit absence but was not associated with non-structural content in meristems. This suggests a combined action of promoting and inhibiting signals originating from leaves and fruit, and involving gibberellins. Nevertheless, these signals act at short distance only since leaf or fruit presence at long distances had no effect on floral induction. Conversely, fruit growth was affected by leaf presence even at long distances when sink demands were imbalanced within the tree, suggesting long distance transport of carbohydrates. We thus clarified the inter-dependence and distance effect among organs, therefore their degree of autonomy that appeared dependent on the process considered, floral induction or fruit growth.

## Introduction

In plants, the determination of organ nature, their development and growth are considered as interdependent. For instance, the position at which flowers develop is linked to the number of nodes developed from the seed (Sachs, 1999). Architectural analyses have revealed a highly structured organization in a wide range of plants, with particular types of organs observed at particular positions and at particular times during ontogenesis (Barthélémy and Caraglio, 2007). This has been demonstrated for instance for the position of reproductive organs in *Quercus ilex* or *Pinus halepensis* (Barthélémy and Caraglio, 2007) or for flower buds along axes in different *Rosaceae* species (Costes et al., 2014). The inter-dependence and differential development of organs within plants are assumed to depend on water, carbohydrates, hormones, mineral nutrients, etc. that are transported within the plants. Both the availability of these resources and the total number of competing organ define a developmental and growth context for each organ depending on its position during plant life span. Among these shared resources, carbohydrates have been particularly studied as they are considered as a main limiting factor for organ growth (Grossman and DeJong, 1995) and as a regulator of the transition between vegetative and reproductive phase in plant life (Rolland et al., 2006). In the particular case of fruit trees, the number, position of fruits, as well as their weight at harvest are dependent on the capability of a given meristem to be floral, then of this flower to fruit set, and finally of a fruit to capture resources for its growth.

In fruit trees, the capability of a shoot apical meristems (SAM) to be floral induced is strongly affected by the presence of fruit during the growing season. A first hypothesis explaining floral induction (FI) inhibition in conditions of high crop load is associated with a competition for carbohydrates between meristems and fruit (Monselise and Goldschmidt, 1982). Besides this “carbon” hypothesis, Chan and Cain (1967) have demonstrated that FI is inhibited by seed development through hormones. This hypothesis was confirmed by experiments on seedless apple and pear cultivars suggesting that seeds may inhibit FI, probably by gibberellins (Dennis and Neilsen, 1999). Gibberellins (GA) are considered among the pathways involved in floral induction control in *Arabidopsis thaliana* (Jung et al., 2017). Their effect is currently considered as inverse in *A. thaliana* and in perennial woody plants. Indeed, GA promotes the transition from vegetative to reproductive development of buds in *Arabidopsis* (Wilson et al., 1992), while it is assumed to inhibit FI in fruit trees such as mango (Nakagawa et al., 2012) and apple (Wilkie et al., 2008). GA12 has been observed as the transported GA form moving within the plant through the vascular system in *Arabidopsis* (Regnault et al., 2015). In the apple tree, GA4 has been assumed to move from fruit to SAM (Ramírez et al., 2004). The involvement of GA in FI control was further confirmed by differential expressions of genes involved in the GA biosynthesis pathway (GA20ox and GA2ox) in SAM of apple trees with heavy or low crop loads (Guitton et al., 2016). This study also suggested a context of carbohydrate starvation, in SAM of trees in high cropping conditions. Therefore, the co-involvement of carbohydrate and hormones in FI control appears as an assumption to further investigate. It implies the involvement of several processes: photosynthesis by leaves, transport from leaves to sinks, including SAM, but also the presence of GA in SAM likely the active forms GA4 and GA1 (Ramírez et al., 2004). Moreover, leaves may have a dual role in FI control since, in addition to being source of carbohydrates, they are also producing FLOWERING LOCUS T (FT) protein, which is transported to the SAM to activate floral induction in many species, including fruit species (Hanke et al., 2007). Nevertheless, it is currently still unclear at which distance the different “signals” originating from fruit and leaves act on FI in SAM.

Regarding carbohydrates, partitioning from sources to sinks is considered as a function of source supply, sink demand and distances between them (Lacointe, 2000). Nevertheless, in fruit trees there is no clear consensus about the impact of distances between sources and sink on carbohydrate allocation and on their consequences on the existing organ growth variability within the trees. Depending on their strength, i.e. the ability of an organ to import assimilate, sinks can use carbohydrates from nearby or distant sources. Carbohydrates can move at short distances, i.e. from non-fruiting to fruiting shoots (Walcroft et al., 2004; Pallas et al., 2018) to sustain fruit growth or at longer distances, i.e. between branches (Palmer et al., 1991; Hansen, 1977). Conversely, authors have suggested that branches can be considered as autonomous (Sprugel et al., 1991). For instance, in shading experiments on walnut, sunlit branches have been observed to grow faster than shaded ones without any allocation of carbon to distant sinks (Lacointe et al., 2004), thus emphasizing the sink strength limitation to long distance transport. This limitation of long distance carbon transport has potential impacts on developmental and growth processes. For instance, part-tree thinning of flower cluster has been shown to enhance branch vegetative growth and floral induction in the thinned tree sides (Palmer et al., 1991; Grossman and Dejong, 1998). Similarly, shoot growth and to some extent starch accumulation in woody organs are impacted by fruit proximity (Berman and Dejong, 2003; Castillo-Llanque and Rapoport, 2011). Moreover, carbon transport and allocation change during a season. Carbon labelling experiments have shown that carbon is allocated from reserves to support new shoot growth in spring (Kandiah, 1979a). At fall, carbon accumulate in leaves moves at long distances to roots to contribute to root growth and storage, before being reallocated to new growth in the next year (Hansen, 1967; Kandiah, 1979b).

In plant, carbon allocation between organs is commonly analyzed through the variations in organ biomass and non-structural carbohydrate (NSC) content. Among the different NSC forms in apple tree, starch, sorbitol, and sucrose are the carbohydrates directly derived from the photosynthetic activity, with sorbitol and sucrose being the mobile forms for carbohydrate transport (Escobar-Gutiérrez and Gaudillère, 1996; Teo et al., 2006). Sorbitol and sucrose are transferred through the phloem to the sinks where they are converted into glucose and fructose (Teo et al., 2006). Starch is commonly stored in reserve organs during the vegetative season. This NSC form is accumulated in reserve organs, where it can be mobilized for regrowth in spring or to buffer source-sink imbalances during the growing season (Sala et al., 2012). Moreover, starch concentration, is directly associated to the ratio between source activity and sink demand (Naschitz et al., 2010; Sala et al., 2012).

In this study, we assumed that distances among organs and availability of resources are involved in both organ development (here floral induction) and growth (here considered as mean fruit weight). Our aim was to investigate the relative contribution of tree carbon balance, source-sink distances and GA availability in SAM on FI and fruit growth. For this, we manipulated within-tree source-sink relationships during two years on “Golden delicious” apple cultivar. We set trees in either high (ON trees) or low (OFF trees) crop loads. On these trees, we reduced by half the number of leaves (sources for both carbohydrates and florigen) or fruit (sources of gibberellins and sinks for carbohydrates). These manipulations were performed at different scales in the trees (shoots, branches and one side of the Y-shape trees) in order to clarify the effect of distances between sources and sinks. Moreover, we considered the within-tree variations in carbon acquisition (leaf photosynthetic activity) and accumulation (NSC concentration) and the gibberellins content in SAM to explore their respective involvement on FI. This study provides (i) new evidence of the likely co-involvement of gibberellins from fruits and signals originated from leaves other than carbohydrates in FI control and (ii) new elements on the debate on organ autonomy with respect to carbohydrate transport in trees.

## Material and Methods

### Plant Material and Growing Conditions

The experiment was carried out from 2016 to 2018 on 10-year-old apple trees (cv. “Golden delicious”). The orchard was located at the SudExpé experimental station in Marsillargues, in the south of France (43◦66′N 4◦18′E). Trees were initially planted in 1998 with “Tentation” cultivar grafted on “Pajam2” rootstock and then top grafted with “Golden delicious” in 2005. The orchard was composed of four rows of 75 trees, each tree composed either of one vertical axis or of two main axes (Y-shape trees) arising five centimeters above the grafting point. Pruning and thinning were applied according to commercial practices before the beginning of the experiment in 2016. During the experimental period, all the trees were irrigated and fertilized to avoid any water or mineral deficiency.

### Varying Source-Sink Relationships by Leaf and Fruit Removal Treatments

In spring 2016, around 65 trees in the orchard were set in OFF conditions by removing all the flowers after full bloom (**Supplementary Material Table S1**). No thinning was performed on the same number of trees, in which complete fruit removal was performed in 2015 to get high crop load in 2016. These trees were then considered as ON trees. In the following year (2017), the cropping status was reversed. In both 2016 and 2017, all the flowers of OFF trees were removed just after full bloom to ensure a crop load equal to zero. To determine the period of FI in our conditions, a specific experiment was carried out. Assuming that the period of irreversible inhibition of FI was between 30 and 70 days after full bloom (DAFB) (Foster et al., 2003, Haberman et al., 2016), young fruit were completely removed on a selection of ON trees, at successive dates during this period. Fruit removal was performed at six dates (one tree per date): 30, 36, 42, 50, 56, and 70 DAFB in 2016 and at four dates in 2017 (37, 43, 50, and 58 DAFB).

**Figure 1:**
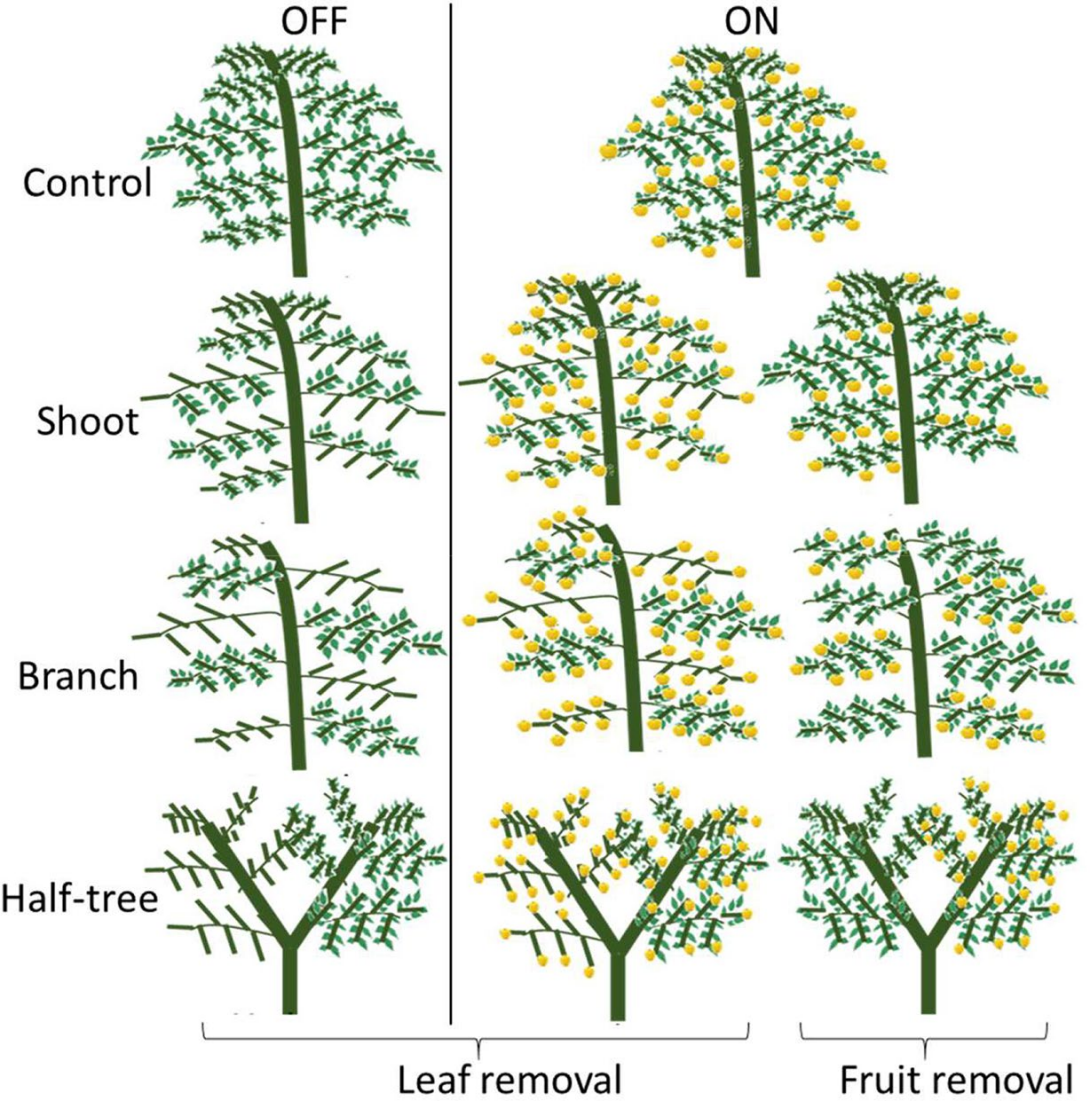
Schematic representation of the leaf and fruit removal treatments.

On the two tree subsets in either ON or OFF conditions, 11 different treatments were set up (three trees per treatment) in springs 2016 and 2017 (**Figure 1**, **Supplementary Material Table S1**). In order to modify fruit and leaves number, half of the leaves or half of the fruit were removed on the trees. Moreover, leaves and fruit were removed in different parts of the trees in order to modify the distances between the remaining leaves and fruit. Leaves and fruit were removed on either half of the shoots, half of the branches or one side of the Y-shape trees. A different set of trees were used in each year for the leaves and fruit removal treatments. New leaves that appeared after the first defoliation in spring were frequently removed throughout the growing season on the trees subjected to leaf removal. Trees not subjected to leaf or fruit removal either on ON or OFF crop load, were considered as controls.

Crop load was estimated in each year by dividing the harvested fruit number per tree by its trunk cross sectional area (e.g. Francesconi et al., 1996). Trunk cross sectional area (TCSA) was estimated each year in autumn after measurements and computed assuming a cylinder shape by measuring in spring the trunk circumference at 10 cm above the grafting zone. For Y-shape trees, crop load was computed for both sides of the tree separately, considering them as mono-axial trees. The tree crop load of Y-Shape trees was then determined, considering this treatment as a combination of two mono-axial trees, by the mean crop load of the two sides of the trees. Crop load in fruit number per TCSA was not computed for leaf removal treatments because it does not account for the impact of such manipulations on tree source-sink ratio.

Another set of trees (called additional trees, **Supplementary Material Table S1**), in either ON or OFF conditions and not subjected to leaf or fruit removal was used to build a reference relationship between tree crop load and mean fruit weight. 69, 103, and 65 trees of the field were considered in 2015, 2016, and 2017 to build this relationship. This reference relationship was then used to evaluate if the impact of local fruit removal treatment was similar to what expected for trees with similar crop load but not subjected to such kind of treatments.

### Development and Growth Variables: Floral SAM Proportion and Mean Fruit Weight At Harvest

The treatment effect on FI proportion in SAM was estimated at full bloom in the spring following treatment in 2017 and 2018 on all the trees including the additional trees and those subjected to sequential thinning in spring. FI proportion was estimated as the ratio of the total number of reproductive buds to the total number of growing buds. This proportion was estimated on six randomly distributed first-order branches per tree in each treatment, considering the leaf or fruit removal conditions (foliated/defoliated, fruiting/non-fruiting, 3 branches per condition). Unfortunately, no data were recorded for the trees subjected to fruit removal at the shoot scale in 2016.

At harvest, in early September of each year, fruit were collected on each treatment. Fruit were sorted by different parts of each tree considering whether they were subjected or not to leaf or fruit removal. All the fruit were collected on each tree except for the treatment performed at the shoot scale for which fruit were collected on two branches per tree, only. Then, each set of fruit was weighted and the mean fruit weight was estimated as the ratio of the total fruit weight to the number of fruit.

### Responses of Leaf Photosynthesis and Starch Content

Leaf photosynthesis and NSC contents were measured on August 2017 (from 119 to 145 DAFB) on fully expanded leaves belonging to short or medium shoots (shorter than 20cm, Costes et al., 2003) and fully exposed to sunlight. Measurements were performed on ON and OFF trees and on foliated parts of the trees with leaf removal treatments and on both fruiting and non-fruiting parts of the trees with fruit removal treatments. Three measurements were performed for each tree and condition (fruit or leaf presence/absence). Measurements were done between 8 and 12 am, with a infra-red gas analyzer (LI-6400, LICOR, Lincoln, Nebraska, USA) under controlled conditions within the measurement chamber known to be non-limiting for photosynthesis (Massonnet et al., 2007) (photosynthetic photon flux density = 1800 µmol m^−2^ s^−1^, relative humidity = RH = 70%, CO_2_ = 400ppm, T = 25°C).

After each photosynthesis measurement, the leaf, the entire annual shoot (called stem) on which the leaf was located, the SAM of this shoot and a 5 cm section of the one-year-old wood supporting it were sampled for measuring their NSC content. Three replicates of all these organs were sampled on each tree and for each condition (fruit or leaf presence/absence). Samples were placed immediately in liquid nitrogen and stored at −20°C for about one week. Then, they were freeze-dried and grinded to fine powders using a ball grinder. Starch concentrations were then determined for all the organs. In SAM, glucose, fructose, sorbitol, and sucrose concentrations were also evaluated. All these analyses were performed following the protocol described in Pallas et al. (2018).

### GA Concentrations in SAM

GA content measurements, were performed on SAM collected on 31 May 2017 (58 DAFB), i.e. at the expected date of FI in short shoots (Foster et al., 2003). SAM were sampled on short to medium shoots that had recently stopped growing and did not formed protecting scars yet. SAM were collected on ON and OFF control trees, on trees subjected to fruit or leaf removal on half of the branches and on trees subjected to fruit removal on one side of Y-shape trees. Nine SAM were collected on each part of the trees (foliated/defoliated, fruiting/non-fruiting) and were gathered together for each tree. All samples were conserved at −80°C before being freeze dried and sent for GA quantification at the Plant Hormone Quantification Service in the Institute for Plant Molecular and Cell Biology (IBMCP), Valencia, Spain. GA are quantified using 30 mg of samples dry weight by Ultra Performance Liquid Chromatography-Mass Spectrometry (UPLC-MS).

Fourteen GA forms produced in the two GAs biosynthesis pathways regulated by the activities of GA20-oxidases (GA20ox), GA3-oxidases (GA3ox) and GA2-oxidases (GA2ox) were investigated (**Supplementary Material Figure S1**). They include bio-active forms (GAs 4 and 1), degradation forms (GAs 51, 34, 29 and 8) and intermediate forms (GAs 12, 15, 24, 53, 44 and 19).

### Statistical Analyses

All statistical analyses were performed with R software (R Development Core team, 2013). We investigated the effects of the combination of (i) the tree treatment (control, leaf removal, and fruit removal), (ii) the scale (tree, shoot, branch, and one side of the Y-shape tree) at which treatments were performed and (iii) the condition within the tree (foliated, defoliated, fruiting and non-fruiting). The effect of all these combinations was tested on photosynthesis, NSC concentrations, GA concentrations and FI proportion, with a one-way ANOVA followed by a Tukey HSD test for pairwise comparisons. Linear models were used for continuous variables and a general linear model of the binomial family was used for FI proportion. For GA and due to the low number of replicates (one per tree and condition), the effect of fruit presence/absence was also tested using Kruskal-Wallis test gathering samples on one hand from control ON and fruiting parts of trees (originated from branch and Y-Shape treatments) and on the other hand from control OFF and non-fruiting parts of trees.

The dataset of additional trees with a large range of crop loads, obtained in 2015, 2016, and 2017 was used to fit an exponential relationship between the tree crop load and the mean fruit weight. The residuals between observed values for a given fruit removal treatment and fruit weight over different crop loads were used to test the impact of local fruit removal treatments under comparable crop load conditions. A linear model between residuals and treatments was then built and the t-value (coefficient of the model divided by its standard error) associated to each treatment was used to assess if the treatment effect was different from 0 (general trend between tree crop load and mean fruit weight).

## Results

### Tree Fruiting Status Between Treatments

Fruit number was not significantly lower for fruit removal treatments compared to control trees in 2016 but the higher TCSA values for these treatments (mainly for shoot and branch treatment) led to a decrease in crop load values (significant for branch treatment only) (**Table 1**). In 2017, a significant decrease in crop load was observed for branch and half tree treatments mainly due to lower fruit number. For fruit removal at the shoot scale crop load was also lower than for control trees but this decrease remained non-significant. For leaf removal treatment TCSA and fruit number values were not significantly different from control trees in both years, although higher fruit number were observed for half-tree treatments (**Table 1**).

**Table 1:** Mean values of fruit number, trunk cross sectional area (TCSA) and crop load estimated as the fruit number per trunk cross sectional area (fruit cm^−2^) for different treatments and scales at which treatments were performed and for the control ON trees of “Golden delicious” apple cultivar in 2016 and 2017.

Tree treatment	Scale	Year					
		2016			2017		
		Fruit number	TCSA (cm²)	Crop load (fruit cm^–2^)	Fruit number	TCSA (cm²)	Crop load (fruit cm^–2^)
Control ON	Tree	366^ab^	27.7^b^	12,1^a^	601^ab^	36.5	20,7^ab^
Leaf removal	Shoot	432^ab^	33.0^ab^		464^b^	34.1	
	Branch	528^ab^	33.7^ab^		591^ab^	36.3	
	Half-tree	655^a^	34.7^ab^		960^a^	25.7	
Fruit removal	Shoot	411^ab^	49.9^ab^	8,5^ab^	581^ab^	37.6	15,9^ab^
	Branch	366^ab^	53.9^a^	6,8^b^	294^b^	35.6	8,2^b^
	Half-tree	279^b^	28.1^b^	9,98^ab^	350^b^	28.7	12,2^b^
Treatment effect		*	**	*	**	ns	**

For each “Half-tree” tree, two TCSA values were considered and corresponded to both sides of trees. Crop load values for “Half-tree” trees was computed as the mean of crop loads on the two sides. Treatment effect was estimated with a one-way ANOVA on three trees for each combination of treatment and scale at which treatment was performed. **Significant at 0.001 < P < 0.01,*significant at 0.01 < P < 0.05 and ns non-significant. A Tukey’s HSD test for pairwise comparisons was made after ANOVA and different letters in a column indicate statistically different values among all treatments.

### Floral Induction in SAM Occurs After Treatment Onset

The complete fruit removal performed sequentially in springs 2016 and 2017 on a subset of ON trees allowed evaluating the date after which the inhibition of FI by fruit presence was no longer reversible. The quantification of FI proportion in the following spring revealed that FI was no longer possible at 70 DAFB (**Table 2**). At that date, FI proportion reached values similar to those of control ON trees. Conversely, when fruit removal was performed before 50 DAFB, FI proportion was close to 100% as observed for OFF control trees, in both years (**Table 2**). Assuming that dates of FI were similar for all the buds in trees, this suggests that FI likely occurred during a short period between 50 and 70 DAFB. This shows that our experimental design was relevant since treatments were performed before 50 DAFB at a date when the SAM fate was not yet determined.

**Table 2:** Proportion of shoot apical meristems (SAM) induced to flower in 2017 and 2018 in “Golden delicious” apple trees subjected to complete fruit removal performed sequentially from 30 to 70 days after full bloom (DAFB), in 2016 and 2017 and for control OFF and ON trees.

Treatment	Floral induction 2017		Floral induction 2018	
	Date of fruit removal 2016	FI proportion	Date of fruit removal 2017	FI proportion
	(DAFB)		(DAFB)	
Control OFF	–	97%	–	100%
Fruit removal	30	95%	–	–
	36	79%	37	98%
	42	70%	43	100%
	50	76%	50	90%
	56	40%	58	56%
	70	18%	–	–
Control ON	–	19%	–	29%

FI proportion was evaluated based on the floral bud proportion, as the ratio of the total number of reproductive buds to the total number of growing buds, on six branches per tree, in the next spring, after the year of treatment.

### FI Proportion Is Affected by Leaf and Fruit Presence and by Their Distance to the SAM

**Figure 2:**
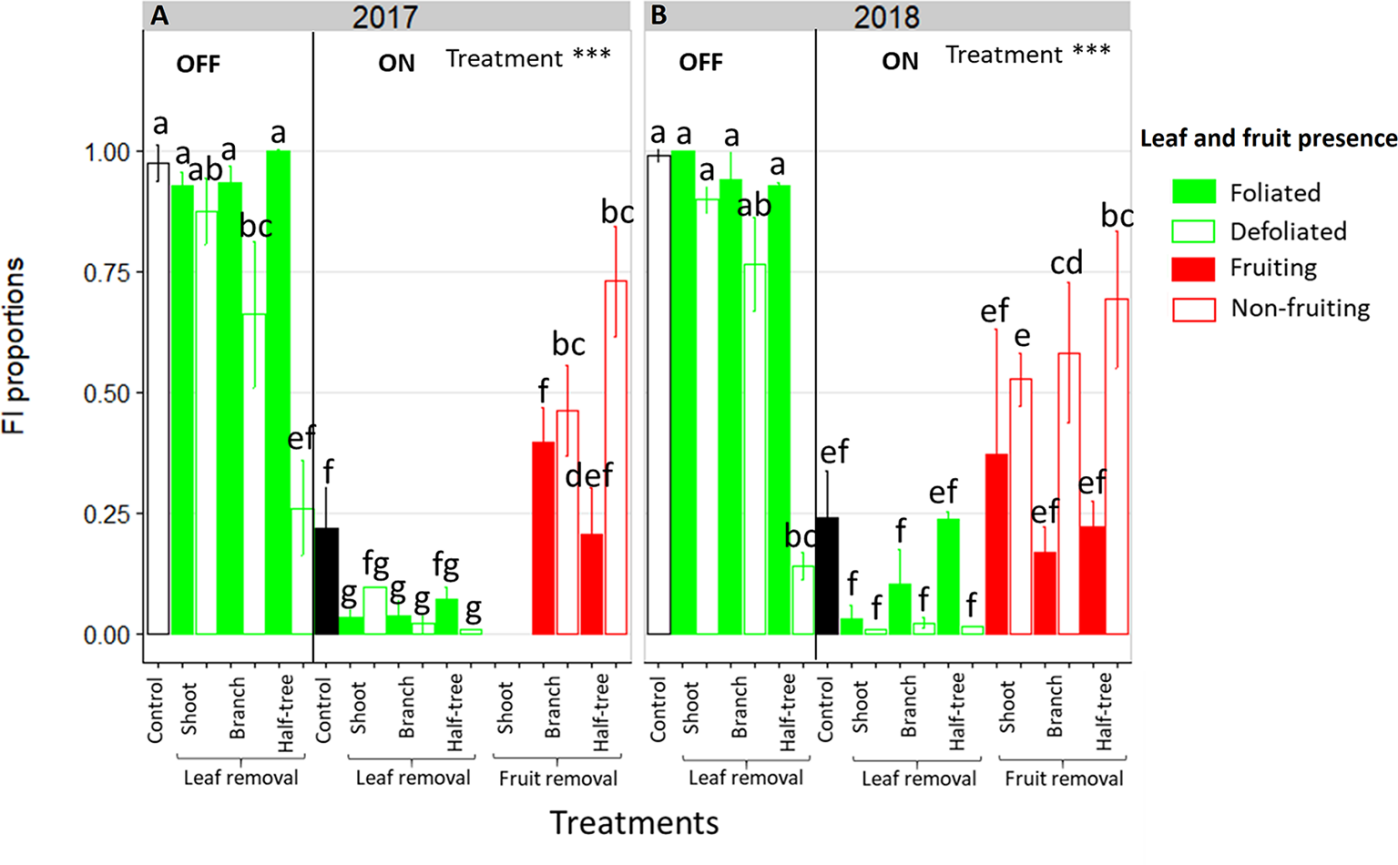
Bar plot representation of FI proportion in ON and OFF “Golden delicious” apple trees for the different treatments in 2017 **(A)** and 2018 **(B)**. Each bar represents the value for one combination of tree treatments and tree scale at which treatments were performed and conditions within the trees (leaf or fruit presence) and lines represent the standard deviation among measurements (3 measurements for each treatment combination). The dataset was fitted with a glm model (binomial family) and treatment effect was assessed with one-way-ANOVA considering all combinations together. *** Significant at P < 0.001. A Tukey’s HSD test for pairwise comparisons was made after the analysis and different letters indicate significant differences among all conditions.

Leaf removal did not impact FI on ON trees, with values close to zero on both foliated and defoliated parts, whatever the scale at which leaf removal was performed (**Figure 2**, right side). In the foliated parts of the defoliated OFF trees FI proportion was similar to the control OFF trees (between 0.9 and 1, **Figure 2**, left side). In contrast, a decrease in FI proportion was observed in the defoliated compared to the foliated parts of trees after leaf removal on half of the branches (−29% and −19% for 2017 and 2018 respectively) or half of the tree (−74% and −63% for 2017 and 2018 respectively). Stronger decrease in the defoliated side of Y-shape trees than on defoliated branches was observed, suggesting an impact of the distances to the remaining leaves on FI. This distance effect was also found by the absence of any significant decrease in FI on the defoliated shoots (−6% and −10% for 2017 and 2018 respectively) of trees subjected to leaf removal at the shoot scale (**Figure 2**). Local fruit removal had also a strong effect on FI proportion within the tree (**Figures 2A, B**, right side). FI proportion was lower in the fruiting than in the non-fruiting parts. Consistently with the distance effect observed after leaf removal treatments, FI proportion increased in non-fruiting parts compared to the fruiting ones when the distances to the remaining fruit increased. In 2018, the increase in FI proportion between fruiting and non-fruiting parts was equal to 50.1% for Y-shape trees while it only reached 29% when fruit removal was performed at the shoot scale.

### Mean Fruit Weight Is Affected by Distances Between Leaves and Fruit

**Figure 3:**
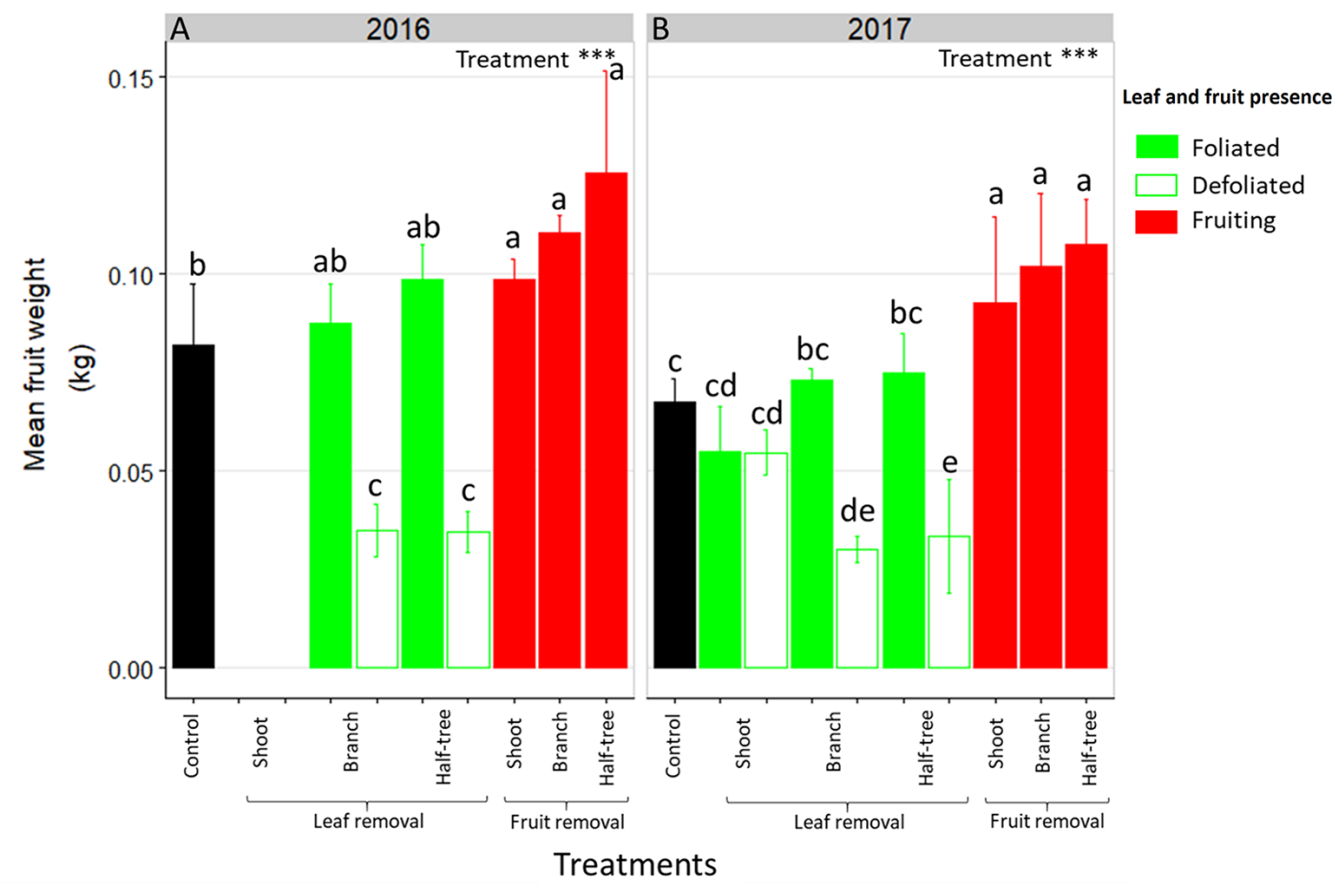
Bar plot representation of mean fruit weight in ON “Golden delicious” apple trees for the different treatment combinations in 2016 **(A)** and 2017 **(B)**. Each bar represents the value for one combination of tree treatments and tree scale at which treatments were performed and condition within the trees (leaf or fruit presence) and lines represent the standard deviation among measurements (3 measurements for each treatment combination). Treatment effect was estimated with a one-way-ANOVA considering all combinations together. *** Significant at P < 0.001. A Tukey’s HSD test for pairwise comparisons was made after the analysis and different letters indicate significant differences among all combinations.

As for FI, mean fruit weight depended on the distances to remaining leaves in trees subjected to leaf removal (**Figure 3**). Indeed, mean fruit weight decreased of 53 and 59% in 2016 in the defoliated parts of trees subjected to leaf removal at the branch scale or on half of Y-Shape trees, respectively compared to the foliated parts. Conversely, this decrease was no longer significant (equal to 2%) when defoliation was performed at the shoot scale. In both foliated and defoliated parts of these trees (defoliation at the shoot scale), mean fruit weight was not significantly different to what observed for control ON trees. Fruit removal increased mean fruit weight compared to control ON trees. Nevertheless, this increase was higher when fruit removal was performed at the branch scale or on one side of Y-Shape trees than after fruit removal at the shoot scale.

**Figure 4:**
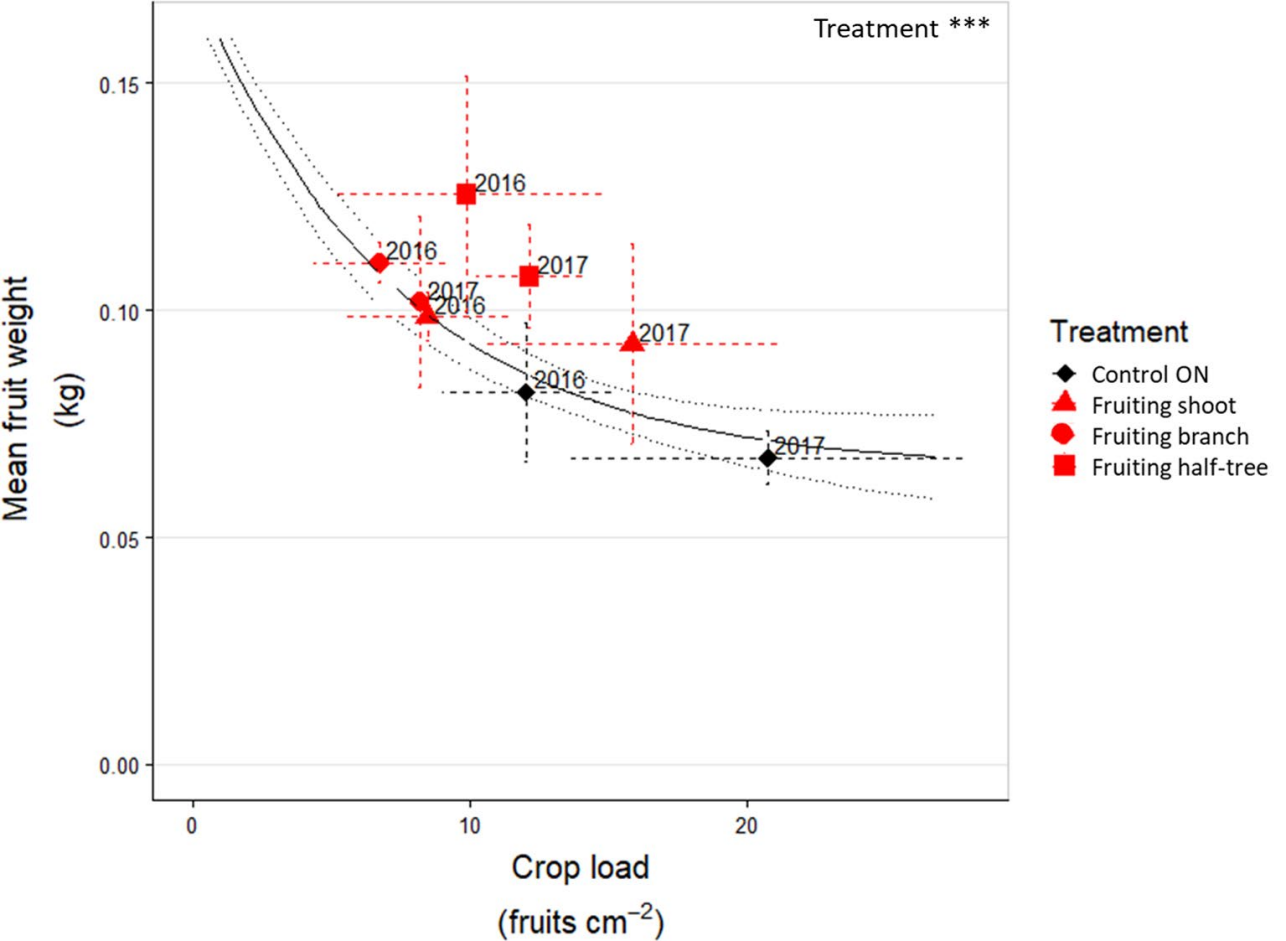
Relationship between mean fruit weight and crop load in ON “Golden delicious” apple trees for control trees and the different fruit removal treatment combinations in 2016 and 2017. Each point represents the value for one combination of tree treatments and tree scale at which treatments were performed and lines represent the standard deviation among measurements (3 measurements for each treatment combination). The continuous black line represents the exponential function fitted on the additional trees dataset (**Supplementary Material Figure S2**). The dotted grey lines represent the deviation interval of the fitted values.

The general trend of mean fruit weight over crop loads, estimated with the additional control trees over three years, displayed a negative relationship (R² = 0.71 between fitted and observed values, **Figure 4** and **Supplementary Material Figure S2**) with highest mean fruit weights equal to around 0.25 kg and lowest ones to 0.08 kg. For the fruit removal treatment, the analysis of residuals to the relationship between crop load and mean fruit weight (**Table 3**) showed that the mean fruit weight in the fruiting shoots (2016) and branches (2016 and 2017) after fruit removal treatments was similar to that of control trees with similar crop load and with a homogeneous distribution of fruit within the tree. A slight increase in residual values (around +0.02 kg) was observed when fruit removal was performed on one side of the Y shape trees in both years or on half of the shoots in 2017. These results suggest that fruit weight was mainly determined by the tree crop load whatever the distance to remaining fruits after fruit removal.

**Table 3:** Mean values of residuals extracted from exponential adjustments between the mean fruit weight (kg) and tree crop loads (**Figure 4** and **Supplementary Material Figure S2**) on “Golden delicious” apple trees.

Tree treatments		Mean fruit weight residuals		
	Scale	Fruit presence	2016	2017
Fruit removal	Shoot	Fruiting	−0.002 (ns)	+0.013 (*)
	Branch	Fruiting	+0.001 (ns)	+0.001 (ns)
	Half-tree	Fruiting	+0.029 (***)	+0.021^a^ (***)

A linear model between residuals and treatments was built and the t-value (coefficient of the model divided by its standard error) associated to each treatment was then used to assess if the treatment effect was different from 0 (general trend between tree crop load and mean fruit weight. ns means non-significant, *and *** means significant at 0.01 < P < 0.05 and at P < 0.001, respectively. The exponential adjustment was fitted on the additional set of trees without any treatment and displaying contrasted crop load in 2015, 2016 and 2017.

Data are presented for different treatments and scales at which treatments were performed.

## Relationships Between Fi, Mean Fruit Weight And Carbon Availability 

**Figure 5:**
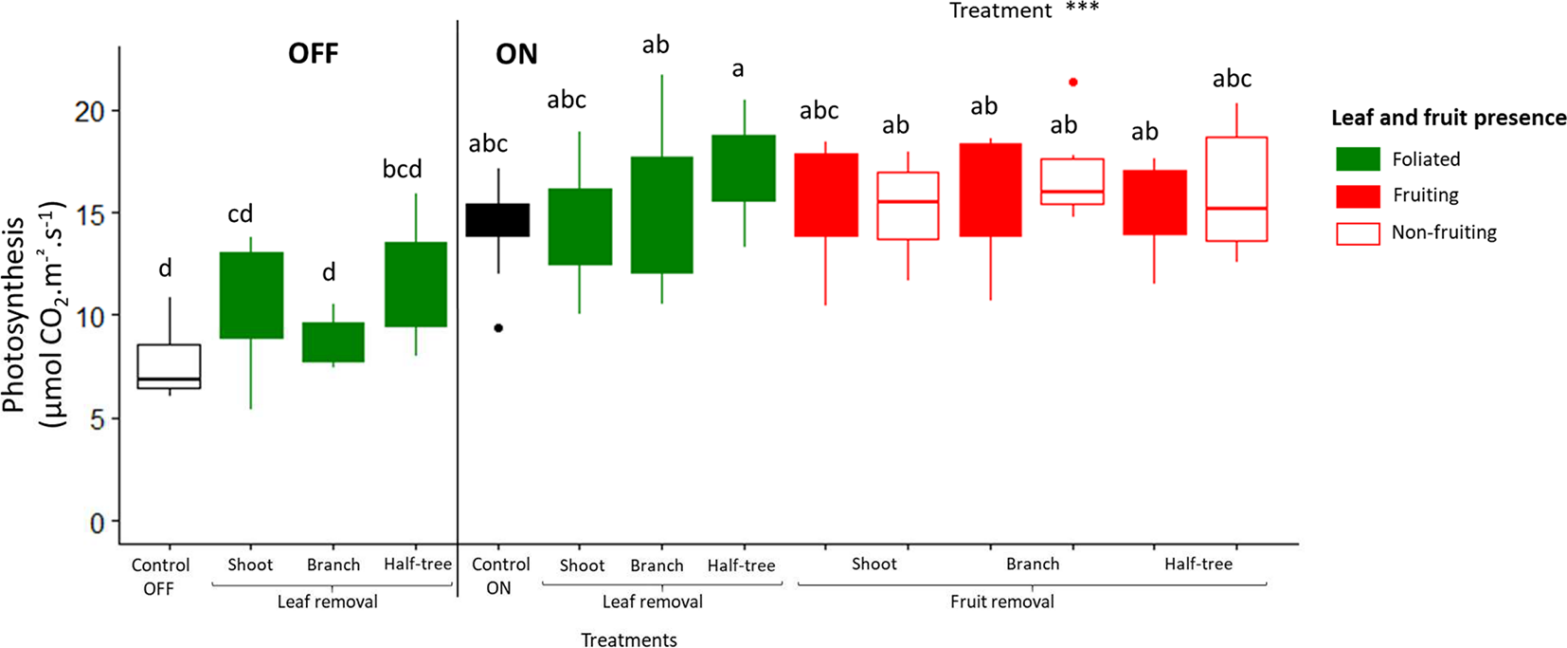
Boxplot representation of leaf photosynthetic activity in August 2017 for “Golden delicious” apple trees for the different treatments (control, leaf removal, and fruit removal), tree scales (tree, shoot, branch, and one side of Y-shape trees) at which treatments were performed and conditions within the tree (foliated, defoliated, fruiting and non-fruiting) for ON and OFF trees. Nine replicates were used for each treatment combination (3 samples × 3 trees). Treatment effect was estimated with a one-way-ANOVA considering all the combinations together. *** Significant at P < 0.001. A Tukey’s HSD test for pairwise comparisons was made after the analysis and different letters indicate significant differences.

Photosynthesis rate was higher for ON trees compared to OFF ones (mean values 7.6 and 14.3 µmol m^−2^ s^−1^ for OFF and ON control trees, respectively; **Figure 5**). Moreover, no significant difference in photosynthetic rates (mean value 15.76 µmol m^−2^ s^−1^ for all fruit removal treatment) was observed between the different fruiting and non-fruiting parts of trees subjected to fruit removal. This suggests that fruit presence stimulated photosynthesis whatever the distances to the fruit.

**Figure 6:**
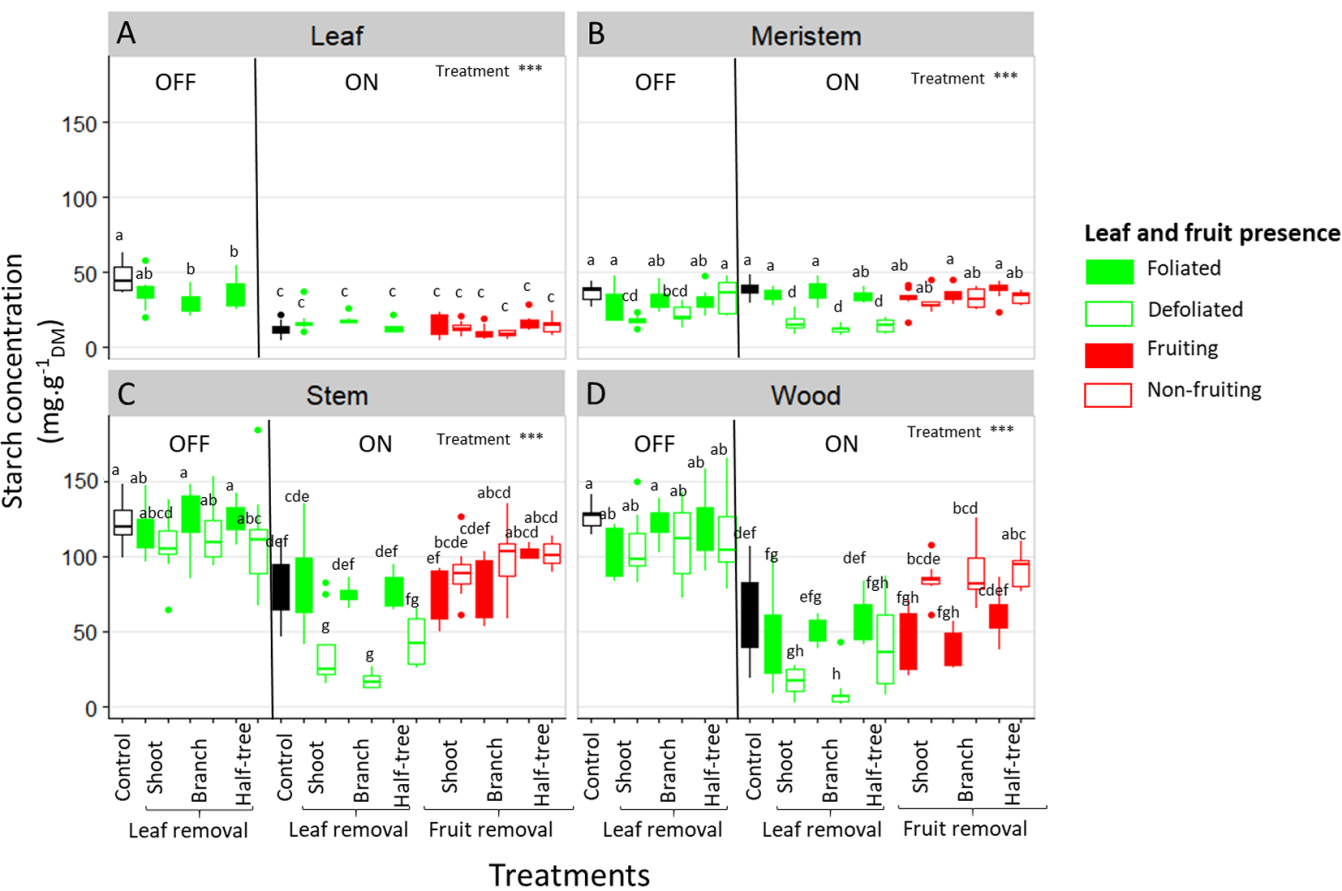
Boxplot representation of starch concentration in the leaves **(A)**, shoot apical meristems **(B)**, stems **(C)** and one-year-old wood **(D)** of ON and OFF “Golden delicious” apple trees for the different treatments, tree scales at which treatments were performed and conditions within the trees (leaf or fruit presence). Nine replicates were used for each treatment combination (3 samples × 3 trees). Treatment effect was estimated with a one-way-ANOVA considering all the combinations together. *** Significant at P < 0.001. A Tukey’s HSD test for pairwise comparisons was made after the analysis and different letters indicate significant differences among all treatments.

Starch concentration varied among organs, with low values in SAM and leaves (**Figures 6A, B**) and higher values in stems and wood (**Figures 6C, D**). Lower values of starch concentration in leaves, stem and wood was observed in ON trees compared to OFF ones whereas no difference between both treatments was observed in SAM (**Figure 6B**). In OFF trees, no impact of defoliation was observed on starch concentrations in SAM, stems and wood (**Figure 6**, left sides), although a decrease in FI proportion was observed in defoliated parts of OFF trees. In contrast, in ON trees, significantly higher starch concentrations were found in the SAM, stems and wood when comparing the leafy parts to defoliated ones (**Figures 6B–D**). For these trees, the effect of leaf removal was similar regardless of the distances to the remaining leaves since no difference was observed between defoliation at the shoot, branch or half tree scale. The effect of fruit removal on starch concentration was observed in wood only, through a greater concentration in the non-fruiting than in the fruiting parts of defoliated shoot, branch and one side of the Y-shape tree treatments (**Figure 6**). No clear impact of the distances to the remaining fruit was observed on starch concentrations in wood, as the decrease in starch content were similar whatever the scale at which fruit removal was performed.

Regarding, the soluble sugars content (sorbitol, sucrose, fructose and glucose) in SAM (**Supplementary Material Figure S3**), sorbitol displayed higher concentrations than the three other sugars. Moreover, treatment effects (fruit and leaf removal) on soluble sugars content in SAM were observed on sorbitol concentration, only after leaf removal treatments on both OFF and ON trees.

### GA9 (Precursor of Active Form) and GA1 (Active Form) Concentrations Decrease in Fruit Presence

In the early-13-hydroxylating pathway (**Supplementary Material Figure S5**), three forms were found in abundance (**Table 4**): GA44 (inactive form), GA1 (active form), and GA8 (degradation form). In the non-hydroxylating GA pathway, maximal GA concentrations were found for GA9 (**Table 4**) which is the last inactive form before GA4 synthesis. Variations in SAM GA contents were observed among all the sampled trees (**Supplementary Material Figure S5**). Nevertheless, differences were significant between SAM from fruiting and non-fruiting parts of trees. GA9 concentration was significantly higher in the SAM collected on the non-fruiting trees or parts of trees (gathering control OFF trees and in non-fruiting branches and sides of Y-shape trees) than in those collected on fruiting trees or parts of trees (gathering control ON trees, fruiting branches and sides of Y-shape trees) (**Table 4**). Even though not significant, higher concentration was observed in the non-fruiting side of the Y-shape tree than in the non-fruiting branches, suggesting a possible effect of distances to the remaining fruit. In addition, a slightly higher but non-significant GA1 concentration was observed in the SAM of non-fruiting parts of the trees than in the fruiting ones. Conversely, GA8 concentration was higher in control ON than in control OFF trees and in fruiting than in non-fruiting branches when fruit removal was performed on half of the branches. Nevertheless, no difference between fruiting and non-fruiting branches was observed for the Y-Shape trees. Finally, leaf removal did not influence any GA concentration.

**Table 4:** Concentrations (ng g^−1^) of GA9, 44, 1, and 8 in shoot apical meristems sampled at 58 days after full bloom on “Golden delicious” apple trees under different treatments (leaf or fruit removal), performed at different scales (branch, half-tree).

Tree treatment	Treatment scale	Fruit presence	Pathways			
			Non-hydroxylating	Early-13-hydroxylating		
			GA9 (ng.g^-1^)	GA44 (ng.g^-1^)	GA1 (ng.g^-1^)	GA8 (ng.g^-1^)
Control ON	Tree	Fruiting	0.43	1.53	2	18.67^a^
Control OFF	Tree	Non-fruiting	2.05	0.86	2.48	2.21^b^
Fruit removal	Branch	Fruiting	0.4	1.22	2.4	15.6^ab^
		Non-fruiting	1.2	1.43	1.9	3.34^b^
	Half-tree	Fruiting	0.21	2.04	1.92	3.83^b^
		Non-Fruiting	2.57	1.41	5.87	4.77^ab^
Leaf removal	Branch	Fruiting (foliated)	0.6	2.64	2.36	3.65^b^
		Fruiting (defoliated)	0.34	1.76	1.88	4.84^ab^
Treatment effect			ns	ns	ns	**
Mean value of fruiting parts		Fruiting	0.39	1.92	2.14	6.98
Mean value of non-fruiting parts		Non-fruiting	1.89	1.42	3.89	4.06
Fruit presence effect			**	ns	ns	ns

Treatment effect on GA concentration was estimated with a one-way ANOVA considering the tree treatment (control, leaf removal, and fruit removal), and the scale (tree, shoot, branch, and one side of the Y-shape tree) at which treatments were performed. **Significant at 0.001 < P < 0.01 and ns non-significant. When significant differences were observed, a Tukey’s HSD test for pairwise comparisons was made and different letters indicate significant differences among treatments. The different conditions were then gathered depending on fruit presence, and the fruit presence effect was estimated with Kruskal-Wallis test. **Significant at 0.001 < P < 0.01 and ns non-significant.

## Discussion

### Relative Roles of Carbohydrates and GA in Flower Induction

Our study investigated the impact of the tree carbon balance on floral induction by exploring the relation between NSC contents in all the organs and SAM status (floral induced or not) after organ manipulations (leaf or fruit removal). After defoliation treatments, the decrease in FI proportion in the defoliated branches and half-side of OFF trees was not associated with any decrease in starch content in all organs including SAM (**Figure 6**). In addition, NSC concentration, whatever the forms (soluble or starch), did not vary between fruiting and non-fruiting parts in all the organs except in wood (**Figure 6**and **S3**), while a decrease in FI was observed in fruiting parts compared to non-fruiting parts. Together these results on trees subjected to leaf or fruit removal suggest that FI is not related to the tree carbon balance and carbohydrate availability in SAM.

Other possible effects of leaf removal independently of the carbon production and primary metabolism (here analyzed through NSC and starch content) could be implicated. Indeed, leaves are likely to be sources of FT protein, considered as the florigen which is transported to SAM where it activates flowering (Corbesier et al., 2007a; Corbesier et al., 2007b; Mathieu et al., 2007; Tamaki et al., 2007). Nevertheless, other carbohydrates than starch and NSC may have role in FI, especially signaling molecules such as trehalose-6-phosphate (T6P, Ponnu et al., 2011; Lastdrager et al., 2014). In *Arabidopsis*, T6P has been shown to affect flowering, by inducing FT production and the expression of flowering-time (SPL) genes in SAM that in turn regulate flowering as a function of plant age (Wahl et al., 2013).

GA4 has been shown to target key flowering genes in SAM, in *Arabidopsis* (Eriksson et al., 2006). In apple tree, GA4 and GA1 may inhibit FI (Ramírez et al., 2001; Ramírez et al., 2004). In the present study, the inactive GA9 preceding GA4 in the non-hydroxylating pathway accumulated in OFF trees, and in non-fruiting branches and side of Y-shape trees (**Table 4**, **Supplementary Material Figure S5**). Nevertheless, no difference in GA4 concentration was found in SAM between fruiting and non-fruiting conditions. In addition, in the hydroxylating pathway, GA1 was slightly higher in non-fruiting tree parts and in OFF trees (**Table 4**, **Supplementary Material Figure S4**) whereas GA8 slightly accumulated in fruit presence. Altogether these results are consistent with the down-regulation of *MdGA2ox* transcripts in OFF trees (or its up-regulation in ON trees), observed in Guitton et al. (2016). They suggest that the last steps of GA catabolism could be less active in absence of fruit (conversely more active in presence of fruit). Therefore, the putative role of GA on controlling FI is supported by our results and previous findings even though their inhibitory or activating effect remains to be clarified. Moreover, further researches would be needed to investigate the ability of GAs, likely produced by seeds (Dennis and Nitsch, 1966), to directly act on SAM FI in the apple tree.

### Response of Floral Induction and Fruit Growth to Changing Source-Sink Distances

In this study, leaf and fruit removal at different scales of plant organization allowed us to clarify the impact of distances between organs on FI and fruit growth. Similar FI proportion was observed between foliated and defoliated shoots and between fruiting and non-fruiting ones (**Figure 2**) when leaf or fruit removal was performed at the shoot scale, thus implying transport at short distances of signals originated from leaves (activators) and fruits (inhibitors). This suggests that shoots can be considered as non-autonomous and prone to exchanges of inhibiting/activating signals.

In contrast, leaf presence in the foliated parts of trees subjected to defoliation at the branch scale or on one side of Y-Shape trees did not promoted FI in the defoliated parts of these trees (**Figure 2**). In that case, distances were too long, possibly for florigen transport, consistently with previous studies having underlined the lack of evidence of FT long distance transport in woody plants (Putterill and Varkonyi-Gasic, 2016). Similarly, FI was only slightly affected by fruit presence at long distance since FI in the non-fruiting branches or sides of Y-Shape trees was slightly lower or even similar to that observed on OFF trees. This suggests that the inhibiting signal produced by fruits may not be transported at long distances in the tree structure or in low quantity, only. GA transport at relatively long distances has been demonstrated but in small annual plants, with GA20 being the mobile form in *Pisum sativum* (Binenbaum et al., 2018) and GA12 the form transported through the xylem in *Arabidopsis thaliana* (Regnault et al., 2015). Interestingly, the different GA forms issuing from the hydroxylated and non-hydroxylated pathways may involve different transporters (Binenbaum et al., 2018). However, studies on the transport of these forms and the distances at which they could be transported in more complex plants such as in fruit trees are still needed.

Fruit weight was also strongly affected by the distances to the remaining leaves after leaf removal (**Figure 3**). As for FI, similar fruit weights were observed between neighboring leafy and non-leafy shoots, when defoliations were performed at shoot scale. This is consistent with previous studies on peach where non-fruiting shoots contributed to fruit growth in nearby fruiting shoots (Walcroft et al., 2004). Conversely, a strong decrease in fruit weight and starch concentrations in all organs were observed on defoliated branches or defoliated parts of Y-Shape trees. This is in accordance with previous studies of carbon labeling on young walnut and peach trees, that have shown limitation of carbon transport at long distance leading to almost complete autonomy of branches even when exposed to source limitation through shading, leaf removal or girdling (Lacointe et al., 2004; Volpe et al., 2008). These results are also consistent with leaf removal effect on fruit growth and reserve accumulation in young fuyu trees and mature *Carpinus*, *Fagus*, and *Tilia* forest trees (Choi et al., 2003; Hoch, 2005).

Conversely, long distance transport of carbohydrates was suggested by the results after fruit removal. Indeed, mean fruit weight in the fruiting parts of trees subjected to fruit removal was similar or even higher to that observed in control trees with a homogeneous crop load (**Figure 4** and **Table 3**). Moreover, starch concentration in stems and leaves of non-fruiting parts of ON trees (**Figure 6**) was lower than that observed in OFF trees suggesting carbohydrate export to the fruiting parts of the trees even at long distances to sustain fruit growth. It is noticeable that a part of the carbon excess produced in the non-fruiting parts was allocated to the reserve organs. This confirms the major role of reserves as an active sink in perennial tress (Silpi et al., 2007). The low NSC content observed in leaves of the non-fruiting parts can be interpreted as resulting from carbon export and prevented photosynthesis inhibition by starch accumulation (Wunsche et al., 2000), thus leading to a similar photosynthesis rate in fruiting and non-fruiting parts of the trees (**Figure 5**). These results are in contradiction with other experimental results showing an impact of the local source-sink conditions on photosynthesis activity (Poirier-Pocovi et al., 2018). Such differences could result from the tree crop load we observed whatever the fruit removal scale (shoot, branch or half tree), ranging from 6.8 to 20.7 fruits cm^–^² (**Table 1**) and corresponding to high crop load conditions, in comparison to previous studies or usual orchard management (Wunsche et al., 2000).

A discrepancy on the distance effects on fruit growth between trees subjected to leaf and fruit removal may appear from our results (**Figure 3**). This apparent discrepancy results from the nature of each treatment. First, leaf removal likely affected the transpiration flux (e.g. Pataki et al., 1998) and may have disturbed the long distance transport of carbohydrates (e.g. Hölttä et al., 2009, Nikinmaa et al., 2013). Second, large within tree source-sink imbalances existed in the trees subjected to fruit removal with the non-fruiting parts displaying low sink demand and large carbohydrate supply and the fruiting parts displaying high sink demand. This imbalance could be the driver of carbon fluxes even at long distance while in the trees subjected to leaf removal fruit sink demand remained high in all the tree parts thus limiting carbon fluxes from the remaining leaves to the distant fruit (Walcroft et al., 2004).

## Conclusion

Our results shows that SAM floral induction is not directly associated to the tree carbon balance nor organ starch content and NSC availability in SAM but more probably to the combination of activating and inhibiting signals originated from leaves and fruit. Having performed leaf and fruit removal at different scales of tree organization provides new clues for understanding the distances at which these signal can act within the tree. At short distances (neighboring shoots), these signals are able to move from sources (leaves and fruit) to sinks (SAM) to act on FI while they cannot reach SAM at longer distances (branches and sides of Y-shape trees). This study suggests that carbohydrates can move at longer distances from branch to branch in condition of high source-sink imbalances within the tree and in absence of any perturbation of the vascular fluxes. Finally, this study brings new considerations on carbohydrate and hormone transports within the fruit trees that can be then integrated in functional structural plant model (e.g., Vos et al., 2009) to simulate floral induction and fruit growth over years.

## Data Availability Statement

All datasets generated for this study are included in the manuscript/**Supplementary Files**.

## Author Contributions

BP, FB, EC, J-JK, and DF conceived and designed the study; BP, FB, SB, DF, and SM collected the data; SB did biochemistry analyses; FB and BP analyzed data and FB, BP, and EC wrote the manuscript; all authors approved the final version.

## Funding

This work was funded by the ANR-DFG Alternapp project and by the PhD grant accorded to FB by INRA BAP department and ITK.

## Conflict of Interest

DF was employed by ITK company. The authors declare that this study received funding from ITK company. ITK was involved in designing the study, data collection and decision to publish. The remaining authors declare that the research was conducted in the absence of any commercial or financial relationships that could be construed as a potential conflict of interest.
