# Supplementary material for: Impact of Within-Tree Organ Distances on Floral Induction and Fruit Growth in Apple Tree: Implication of Carbohydrate and Gibberellin Organ Contents
Source: Front Plant Sci. 2019 Oct 21;10:1233. doi: 10.3389/fpls.2019.01233 (PMC6816281; doi:10.3389/fpls.2019.01233)
Supplement: Supplementary file 1 [file DataSheet_1.pdf]

Supplementary material

Table S1. Overview of the different treatments, the number of tree replicates used and the main objectives of each manipulation

Treatments	Crop status	Year	Tree replicates	Objective
Additional trees	OFF to ON	2015	69	Build a relationship between crop load and mean fruit weight
		2016	103	
		2017	65	
Fruit removal at different dates	ON	2016	6	Estimate FI timing
		2017	4	
Control	ON	2016	3	Compare the trees subjected to leaf and fruit removal to non-modified conditions
		2017	3	
	OFF	2016	3	
		2017	3	
Leaf removal at different scales	ON	2016	3	Evaluate the effect of leaf removal and distances on floral induction and fruit weight
		2017	3	
	OFF	2016	3	
		2017	3	
Fruit removal at different scales	ON	2016	3	Evaluate the effect of leaf removal and distances on floral induction and fruit weight
		2017	3	

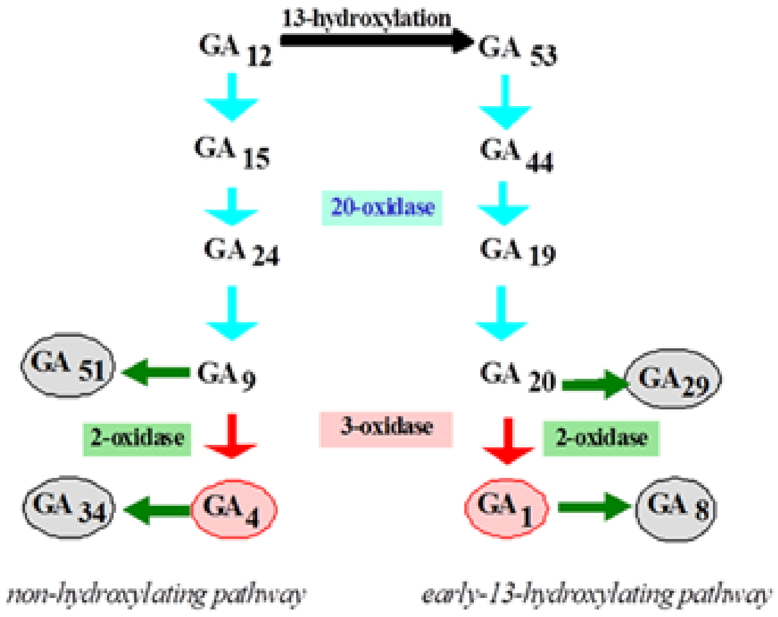

Figure S1. Schematic representation of the gibberellin biosynthesis pathways

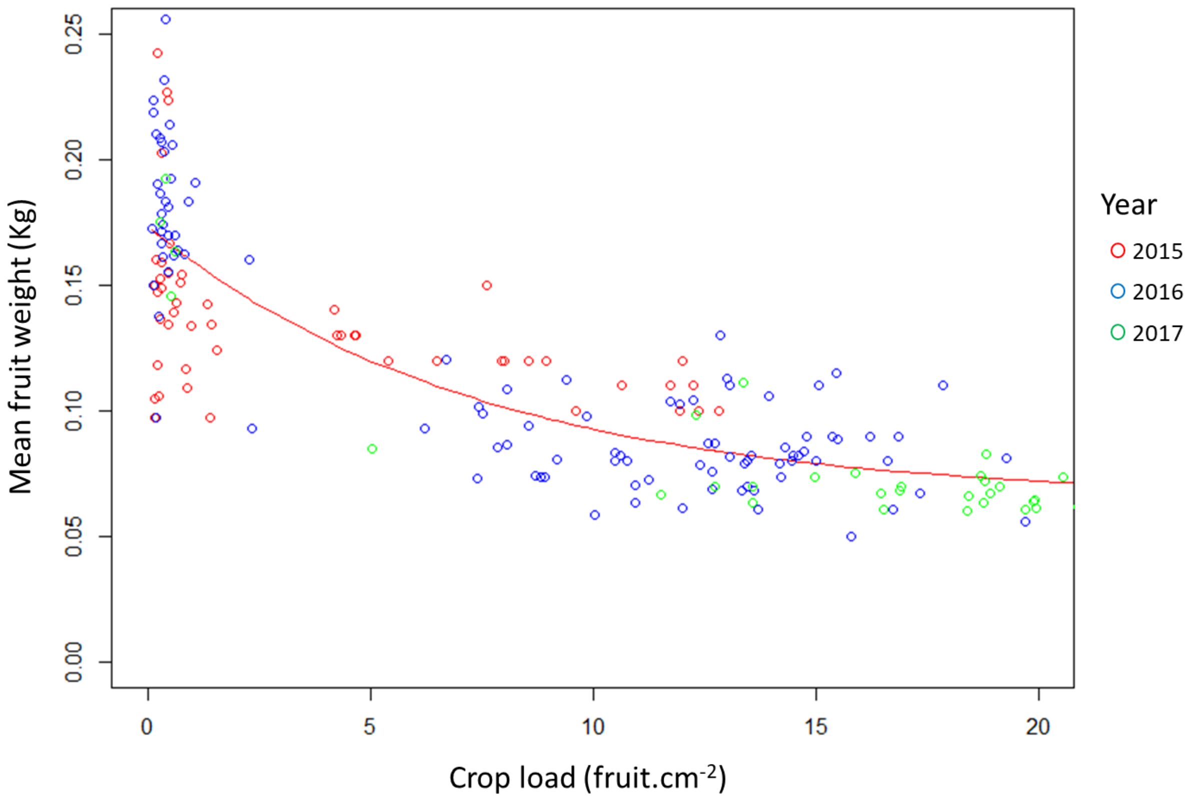

Figure S2. Relationship between crop load and mean fruit weight estimated on the additional ' Golden Delicious' apple control trees in 2015, 2016 and 2017. The continuous line represents the result of the exponential adjustment over the whole dataset (Y= 0.5 × exp (-0.2 × x) + 0.06). R^2 = 0.71 between fitted and observed values.

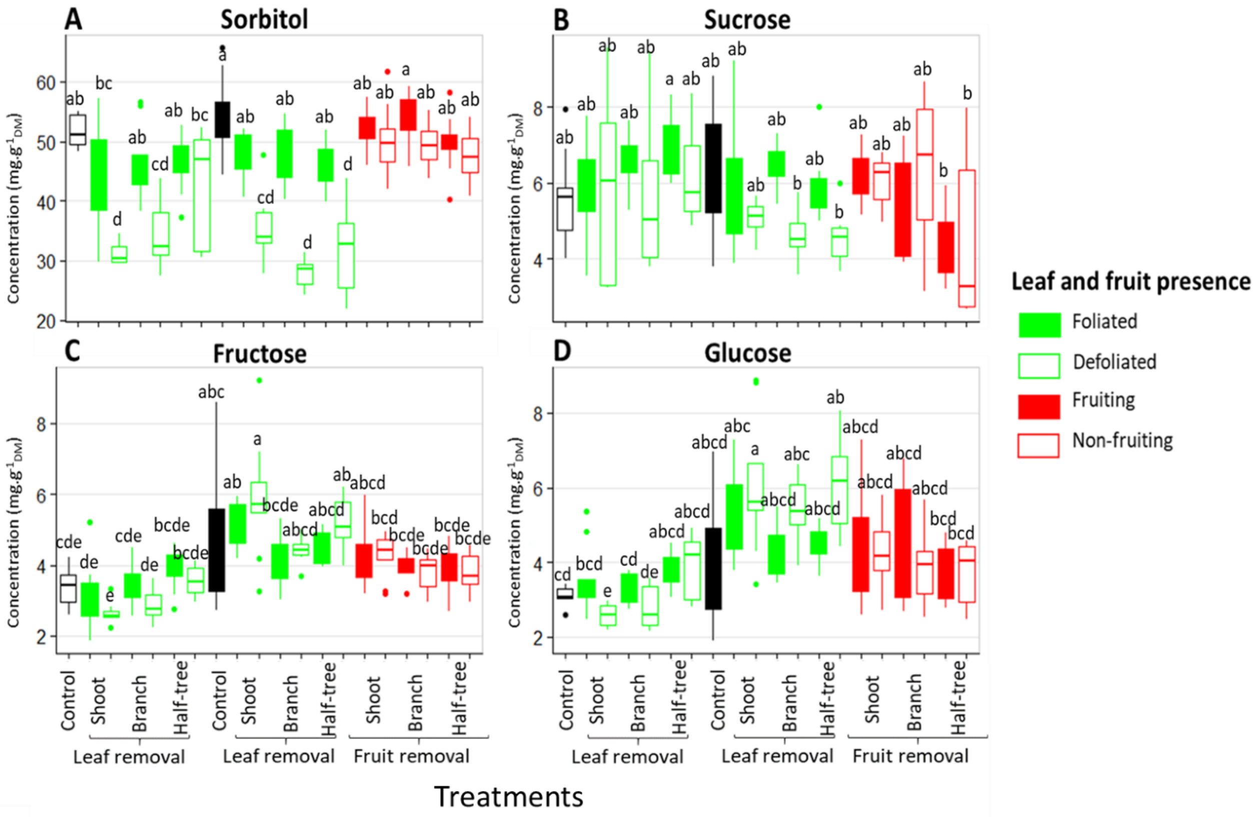

Figure S3. Boxplot representation of sorbitol (A), sucrose (B), fructose (C) and glucose (D) concentration in shoot apical meristems of 'Golden Delicious' apple trees for the different combinations of tree treatment, tree scale at which treatments were performed and conditions (leaf or fruit presence) within the trees. Nine replicates were used for each treatment combinations (3 samples × 3 trees). Treatment effect was estimated with a one-way-ANOVA considering all the combinations together. *** significant at P<0.001. A Tukey's HSD test for pairwise comparisons was made after the analysis and different letters indicate statistically different values among all treatments.

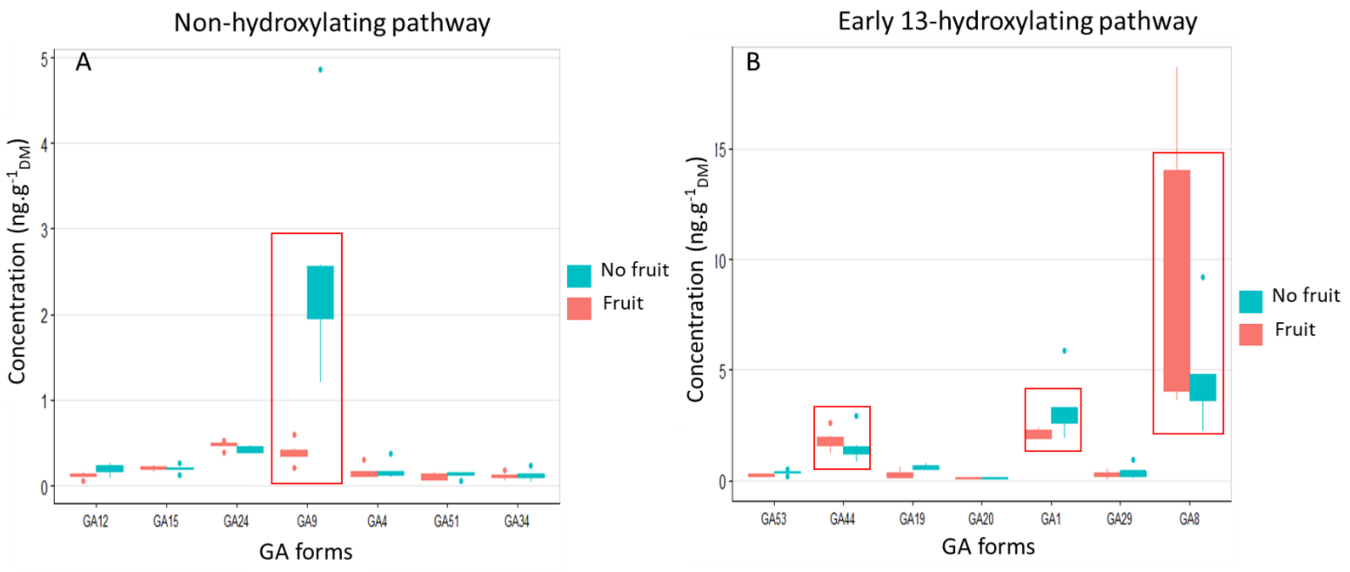

Figure S4. Boxplot representation of concentrations of all gibberellin forms (ng.g^{-1}) from the non-hydroxylating (A) and the early-13-hydroxylating (B) pathways in shoot apical meristems of 'Golden Delicious' apple with or without fruits at 58 days after full boom. Samples were gathered according to fruit presence at the base of the shoot bearing the shoot apical meristem whatever the treatment to which trees have been subjected.

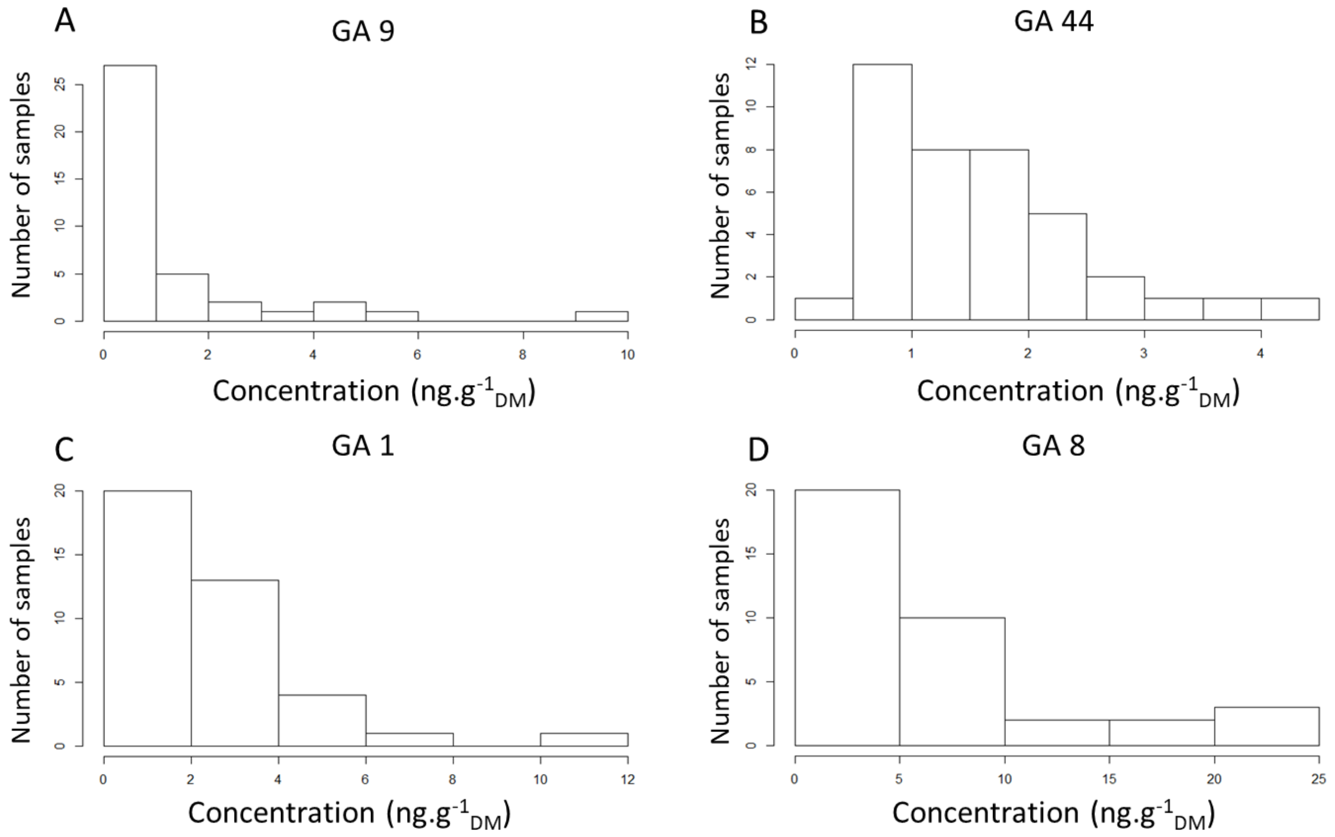

Figure S5. Distribution of concentrations of GA9 (A), GA44 (B), GA1 (C) and GA8 (D), in shoot apical meristems of 'Golden Delicious' apple trees for all the studied treatments at 58 days after full bloom
