# Supplementary material for: Impact of Within-Tree Organ Distances on Floral Induction and Fruit Growth in Apple Tree: Implication of Carbohydrate and Gibberellin Organ Contents
Source: Front Plant Sci. 2019 Oct 21;10:1233. doi: 10.3389/fpls.2019.01233 (PMC6816281; doi:10.3389/fpls.2019.01233)
Supplement: Supplementary file 2 [file DataSheet_2.pdf]

Description of the dataset used for the preparation of the manuscript "Impact of within-tree organ distances on floral induction and fruit growth in apple tree: implication of carbon and gibberellin organ contents".

**CropLoad_MFW- Worksheet:** The dataset includes total fruit weight and number, mean fruit weight, trunk cross-sectional area and crop load in 2016 and 2017 at harvest, for the different treatments and within-tree conditions (leaf/fruit presence). Data were also presented for "control additional trees" in 2015, 2016 and 2017.

**FI:** The dataset includes the number of vegetative and reproductive buds (Total_Number_V, Total_Number_I, respectively) recorded on four branches per tree in spring 2017 and 2018 after full bloom, for the different treatments and within-tree conditions (leaf/fruit presence).

**GA.csv:** The dataset includes information on GA concentrations in shoot apical meristems of *'Golden delicious'* apple cultivar collected in 2017. GA12, GA15, GA24, GA9, GA51, GA4, GA34, GA53, GA44, GA19, GA20, GA29, GA1 and GA8 were quantified for the different treatments and within-tree conditions.

**NSC.csv:** The dataset includes glucose, fructose, sucrose, sorbitol and starch concentration collected in 2017 on leaves, stem, wood and meristems for the different treatments and within-tree conditions.

**photosynthesis.csv:** The dataset includes photosynthesis activity measurements in 2017 for the different treatments and within-tree conditions.
